# Using Naturalistic Driving Data to Predict Mild Cognitive Impairment and Dementia: Preliminary Findings from the Longitudinal Research on Aging Drivers (LongROAD) Study

**DOI:** 10.3390/geriatrics6020045

**Published:** 2021-04-23

**Authors:** Xuan Di, Rongye Shi, Carolyn DiGuiseppi, David W. Eby, Linda L. Hill, Thelma J. Mielenz, Lisa J. Molnar, David Strogatz, Howard F. Andrews, Terry E. Goldberg, Barbara H. Lang, Minjae Kim, Guohua Li

**Affiliations:** 1 Department of Civil Engineering and Engineering Mechanics, Columbia University, New York, NY 10027, USA; sharon.di@columbia.edu (X.D.); rongyes@alumni.cmu.edu (R.S.); 2 Department of Epidemiology, Colorado School of Public Health, University of Colorado Anschutz Medical Campus, Aurora, CO 80045, USA; Carolyn.DiGuiseppi@cuanschutz.edu; 3 Transportation Research Institute, University of Michigan, Ann Arbor, MI 48109, USA; eby@umich.edu (D.W.E.); ljmolnar@umich.edu (L.J.M.); 4 Center for Advancing Transportation Leadership and Safety (ATLAS Center), Ann Arbor, MI 48109, USA; 5 School of Public Health, University of California San Diego, La Jolla, CA 92093, USA; llhill@ucsd.edu; 6 Department of Epidemiology, Columbia University Mailman School of Public Health, New York, NY 10032, USA; TJM2141@cumc.columbia.edu; 7 Center for Injury Science and Prevention, Columbia University Irving Medical Center, New York, NY 10032, USA; 8 Bassett Research Institute, Cooperstown, NY 13326, USA; david.strogatz@bassett.org; 9 Department of Psychiatry, Columbia University Vagelos College of Physicians and Surgeons, New York, NY 10032, USA; Howard.Andrews@nyspi.columbia.edu (H.F.A.); teg2117@cumc.columbia.edu (T.E.G.); 10 Department of Anesthesiology, Columbia University Vagelos College of Physicians and Surgeons, New York, NY 10032, USA; BL2309@cumc.columbia.edu (B.H.L.); mk2767@cumc.columbia.edu (M.K.)

**Keywords:** aging, Alzheimer’s disease and related dementias, artificial intelligence, dementia, driving patterns, machine learning, mild cognitive impairment, naturalistic driving study, random forests, screening

## Abstract

Emerging evidence suggests that atypical changes in driving behaviors may be early signals of mild cognitive impairment (MCI) and dementia. This study aims to assess the utility of naturalistic driving data and machine learning techniques in predicting incident MCI and dementia in older adults. Monthly driving data captured by in-vehicle recording devices for up to 45 months from 2977 participants of the Longitudinal Research on Aging Drivers study were processed to generate 29 variables measuring driving behaviors, space and performance. Incident MCI and dementia cases (n = 64) were ascertained from medical record reviews and annual interviews. Random forests were used to classify the participant MCI/dementia status during the follow-up. The F_1_ score of random forests in discriminating MCI/dementia status was 29% based on demographic characteristics (age, sex, race/ethnicity and education) only, 66% based on driving variables only, and 88% based on demographic characteristics and driving variables. Feature importance analysis revealed that age was most predictive of MCI and dementia, followed by the percentage of trips traveled within 15 miles of home, race/ethnicity, minutes per trip chain (i.e., length of trips starting and ending at home), minutes per trip, and number of hard braking events with deceleration rates ≥ 0.35 g. If validated, the algorithms developed in this study could provide a novel tool for early detection and management of MCI and dementia in older drivers.

## 1. Introduction

As aging of the US population accelerates, the number of older drivers continues to rise. According to the US Census Bureau, there were over 49 million older adults (aged 65 years and older) in the United States in 2016, accounting for 15% of the population [1]. The number of older adults with a driver’s license in the United States is expected to increase from 42 million (or 85% of the older adult population) in 2016 to 63 million in 2030 [2]. While driving allows older adults to meet their mobility needs and to stay independent, age-related functional declines, medical conditions, and side effects of medications can compromise driving abilities and lead to heightened crash risk. In addition, atypical changes in driving behaviors may be early signals of cognitive function declines and dementia. To determine whether a recent history of unsafe driving was associated with cognitive impairment, Ott et al. [3] recorded traffic violations and crashes in the previous 3 years for middle-aged and older patients of an outpatient memory clinic who were cognitively normal or diagnosed with mild cognitive impairment (MCI) or Alzheimer’s disease (AD). In addition to the diagnostic categories, all study participants were classified according to levels of brain amyloid deposits. A significant positive association between the history of crashes and violations with amyloid brain burden was observed at levels below the usual threshold corresponding with moderate to frequent amyloid plaques. In a series of studies based on driving data, Roe and colleagues [4,5,6,7] assessed associations between driving difficulties and AD biomarkers in older adults rated as cognitively normal based on a Clinical Dementia Rating score of 0. Their initial data indicated that levels of brain amyloid burden and cerebrospinal fluid (CSF) biomarkers of neurofibrillary tangles were positively correlated with the number of driving errors during a 12-mile parking lot and road test [5,6]. By repeating the road test annually over a 3.5-year period for the same cohort, Roe et al. [4] and Babulal et al. [8,9,10] showed that the CSF biomarkers predicted time to the participant’s driving test being rated as marginal or a failure. It is worth noting that amyloid biomarkers appear to be associated with driving performance but not with global cognitive test scores, implying that the assessment of driving may be a useful strategy for the early detection of cognitive declines.

Several studies have demonstrated that atypical changes in driving performance and driving behaviors could be detected in older drivers with preclinical AD [4,5,6] and early-stage dementia [11], and that these changes may progress throughout the trajectory of AD [4,5,6,7,12]. In recent years, naturalistic driving study designs have been used for understanding driving behaviors in older adults with preclinical AD [7] and early-stage dementia [11]. Reported changes in older drivers with preclinical AD or early-stage dementia include declines in driving performance, such as increased incidence of getting lost in traffic [11], increased risk of failing a driving test [5,6] and reduced spatial navigation ability [13], and atypical driving behaviors, such as decreased driving exposure (e.g., fewer driving trips, driving days, driving destinations, nighttime driving and rush-hour driving) [7], restricted driving space (e.g., less freeway driving and more driving within 5–10 miles of home) [7,11], and reduced unsafe driving behaviors (e.g., fewer hard braking events and speeding events) [10].

While these naturalistic driving studies help to link driving behavior changes to the risk of MCI/dementia, they are largely limited to piloting data with small sample sizes and short follow-up durations. The AAA Longitudinal Research on Aging Drivers (LongROAD) project is the largest naturalistic driving study of older drivers in the United States [14]. Using preliminary data from the LongROAD project and machine learning techniques, we assessed the utility of objectively measured driving variables in predicting MCI and dementia in older adults. If confirmed, our findings may help to improve early detection and management of MCI and dementia.

## 2. Materials and Methods

### 2.1. LongROAD Study

The LongROAD study is a multisite prospective cohort study of 2990 active drivers aged 65 to 79 years at the time of enrollment. The LongROAD study includes five data collection sites: Ann Arbor, MI; Baltimore, MD; Cooperstown, NY; Denver, CO; and San Diego, CA. Eligibility criteria were established to ensure that study participants were relatively healthy, active drivers aged 65–79 years at the time of enrollment, who would likely be available to be assessed annually through the duration of the study. Among those excluded from the LongROAD study were drivers with Six-Item Screener score < 4, having significant cognitive impairment or being diagnosed with degenerative medical conditions, such as AD, Huntington’s disease, and Parkinson’s disease [14].

The data used in this study spanned the time period from August 2015 through March 2019. Naturalistic driving data were available for 2977 participants, among whom 33 were newly diagnosed with MCI and 31 with dementia up to April 2019. These incident MCI and dementia cases were ascertained from the review of participants’ medical records and the annual interviews [14]. As dementia is a progressive disease and follow-up interviews were conducted annually, it was not possible to delineate the month when the conversion from MCI to dementia occurred. Thus, we classified the MCI/dementia status as a binary variable (yes/no).

The driving behavior profile contains 29 variables that were aggregated monthly and derived from the in-vehicle recording device “DataLogger” (Danlaw, Inc., Novi, MI, USA). Their definitions and the statistics are detailed in Table 1.

### 2.2. Monthly Record Classification

To fully utilize all time-dependent driving data, we treated each monthly record as an independent data point. The data point sizes of health and MCI/dementia were 89,380 and 1063, respectively, with a total of 90,443 data points. Each data point, x, included 33 covariates (i.e., 4 demographic variables and 29 driving variables). The disease status, y, was defined as 0 if healthy or 1 if MCI/dementia. We aimed to develop a classifier to predict the disease status y using data point x.

### 2.3. Five Classification Models

A robust classification technique that involves building multiple decision trees, random forests (RFs) were used to classify the disease status for a given data point. All the analyses were performed in the R environment with Version 1.3.1056.

For each model, we tried five groups of covariates, one with age only, one with demographic characteristics (i.e., age, sex, race/ethnicity, and education) only, one with driving variables only, one with age and driving variables, and one with demographic characteristics and driving variables. Building upon these groups of covariates, we quantitatively assessed the relative contributions of age and other demographics and driving variables to the RF model performance in classifying disease status.

### 2.4. Random Forests

We used RFs for 3 reasons: (1) RF is a versatile and powerful ensemble learning classifier capable of fitting complex datasets; (2) RF is more computationally efficient than other classification models, such as artificial neural networks; and (3) RF provides highly interpretable results through importance rankings of covariates.

#### 2.4.1. Performance Metrics

To evaluate the performance of the classification models on test datasets, we focused on 3 metrics: precision, recall, and F_1_ score. These metrics measure different aspects of the performance of the classifier and are better suited to different outcomes. For example, if our goal is to train a classifier to detect those with potential dementia symptoms in order to intervene at an early stage and allow them to be treated in time, then a classifier that captures almost all participants exhibiting early signs of dementia (i.e., high recall or sensitivity) at the expense of some false positives is preferable. If the goal is to train a classifier to inform those who are likely to develop dementia without misidentifying too many of those who are likely not, then a classifier that minimizes the false positives (i.e., a healthy participant is identified for dementia) and has high precision (i.e., positive predictive value) is preferable. If the goal is to demonstrate the feasibility of using driving variables to detect MCI and dementia, then recall and precision should be balanced. The F_1_ score, which is the harmonic mean of recall and precision, was used to measure the overall performance of the classifier. A classifier with a high F_1_ score could be valuable for early detection of MCI/dementia as well as for improving driving safety. In addition, we calculated the area under the receiver operating characteristic curve (AUC) as a measure of validity for model discrimination.

To train RF classifiers, we needed to first divide the dataset into a test dataset and a training dataset.

#### 2.4.2. Test Data Selection

We randomly selected 77 healthy and 102 MCI/dementia data points as the test or validation data, which were used to evaluate RF classifiers with performance metrics. We deliberately selected more MCI/dementia points than healthy points to ensure adequate data for assessing the accuracy of the RF classifiers.

#### 2.4.3. Training Data Rebalance

The training data were the remaining data points after the selected test data were removed from the total dataset. As the total dataset was highly imbalanced (with many more healthy data points than MCI/dementia points) and could cause training bias, we needed to first rebalance the training data.

The data were highly skewed from both the perspective of drivers and monthly records. Of the 2977 participants included in this study, only 64 (2.1%) developed MCI/dementia during the follow-up. Of the 90,443 monthly data records, 89,380 were healthy points (labeled as 0) and 1063 were MCI/dementia points (labeled as 1). The monthly data had a healthy versus MCI/dementia class ratio of 84:1. In other words, the MCI/dementia monthly data accounted for only 1.2% of the entire dataset.

To mitigate the imbalance issue, we applied the synthetic minority oversampling technique (SMOTE) to oversample the dementia data points such that the amount of dementia class was comparable to that of the healthy class without compromising the total sample size too much. We oversampled the dementia data points and generated synthetic MCI/dementia samples using existing MCI/dementia data while undersampling the healthy points to make the healthy–MCI/dementia class ratio close to 0.95:1. This was carried out using the R function “SMOTE” in the package “DMwR”. After rebalancing, the training dataset contained 39,401 records with 19,220 healthy and 20,181 MCI/dementia records.

#### 2.4.4. Parameter Tuning

Parameter tuning helps to control the training process and improve the result. The tuned parameters were divided into 2 types: RF parameters and prediction cutoff threshold. In RF training, the key parameter was “mtry” (i.e., number of variables randomly collected to be sampled at each split node). In the prediction stage, RF could predict a crisp class label or a continuous probability score. In the latter case, an optimal “decision threshold” would be crucial to convert from a probability score to a crisp class label. For a balanced dataset, 0.5 is often used as the optimal threshold. However, for imbalanced classification, the optimal threshold needs to be tuned.

To tune these parameters, 10-fold cross-validation was employed. In other words, the training set was split into 10 folds where a RF model was trained with nine folds and evaluated on the remaining fold. A grid search was performed on a combination of parameter values and the optimal ones were selected based on the highest F_1_ score.

## 3. Results

### 3.1. Model Comparison

Table 2 displays the performance metrics and confusion matrix of the five RF models evaluated on the test dataset. Each row represents one classification model with one particular group of covariates. Models 1, 2, 3, 4, and 5 refer to the ones with the covariate of age only, demographic characteristics (i.e., age, sex, race, and education) only, driving variables only, both age and driving variables, and both demographic characteristics and driving variables, respectively.

Model 5 with combined demographic characteristics and driving variables achieved the highest F_1_ score of 0.88, followed by Model 4, with a F_1_ score of 0.81. Contrasting Model 4 with Model 1 revealed that adding driving variables increased the validity of the MCI/dementia prediction from 0.11 based on age only to 0.81. Model 1 (with age only) achieved a perfect precision (1.00) but a poor recall (0.06), while Model 3 (with driving variables only) achieved a fair recall (0.56) and a moderate precision (0.79). Combining age and driving variables, Model 4 demonstrated both a high precision (0.89) and a moderate recall (0.74). Likewise, contrasting Model 5 to Model 2 revealed that adding driving variables increased the validity in predicting MCI/dementia from 0.29 to 0.88.

### 3.2. Feature Importance Ranking

**Figure 1:**
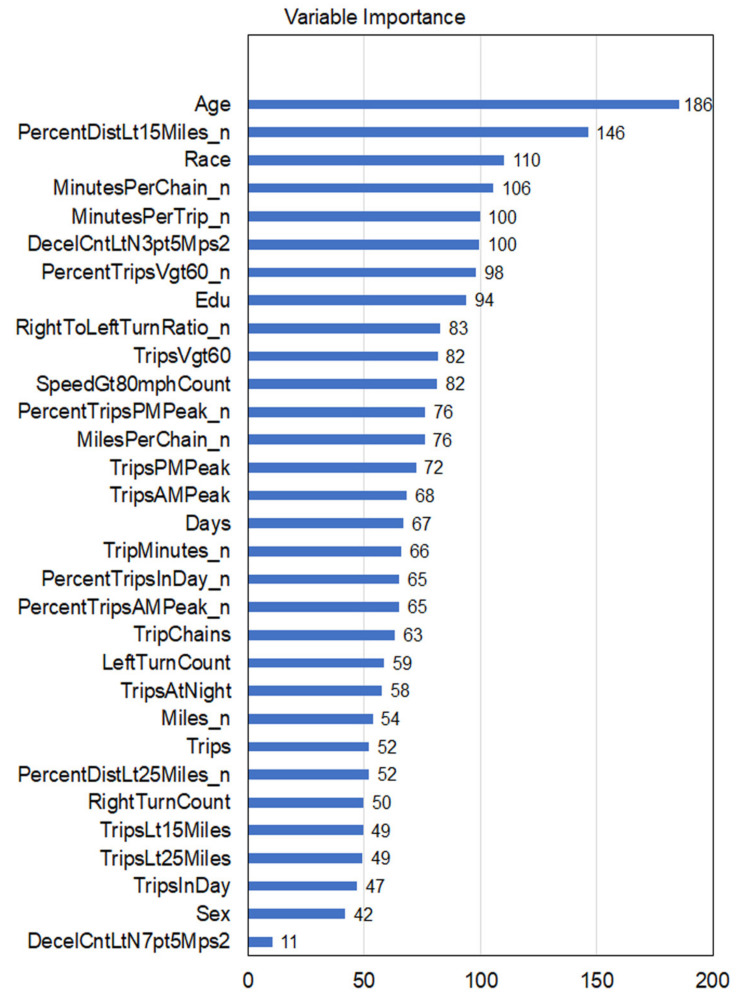
Feature importance ranking.

To assess the relative importance of each covariate in building RF classifiers, we plotted the feature importance ranking in terms of “mean decrease accuracy”, shown in Figure 1. Mean decrease accuracy measures a covariate’s importance by quantifying how much the tree nodes that use the covariate at a split reduce predicted accuracy on average. Age was the most important feature in developing the classifiers. Among driving variables and other demographics, the top five covariates were: percent of driving distance less than 15 miles from home (PercentDistLt15Miles_n), race/ethnicity (Race), minutes per trip chain (MinutesPerChain_n), minutes per trip (MinutesPerTrip_n), and number of hard braking events with deceleration rates ≥ 0.35 g (DecelCntLtN3pt5Mps2). These covariates were related to different aspects of driving: “PercentDistLt15Miles_n” reflecting the driving space, “MinutesPerChain_n” and “MinutesPerTrip_n) representing driving time duration, and “DecelCntLtN3pt5Mps2” indicating unsafe driving maneuver.

## 4. Discussion

We trained five RF classifiers with two groups of covariates, demographic characteristics (i.e., age, sex, race/ethnicity and education) and driving variables. The one with both demographic characteristics and driving variables showed an overall predictive validity of 88%, implying that using both the knowledge of basic demographics and driving behavior could accurately predict if one has MCI/dementia. However, even using driving variables only achieved a reasonably good predictive performance, especially if our goal is to identify those exhibiting early signs of MCI/dementia.

This study is among the first to assess the feasibility of using a large amount of naturalistic driving data and machine learning techniques to detect MCI/dementia. The usefulness of driving data in MCI/dementia classification could have important implications for the screening and early treatment of MCI and dementia. Early detection of MCI and dementia may also help improve driving safety for older adults. It is worth noting that this study assessed the value of driving behavior in predicting MCI/dementia rather than the influence of MCI/dementia on driving behavior as shown in previous studies [3,4,5,6,7,8,9,10,11,12,13].

The novelty of this study also lies in the application of machine learning techniques (i.e., random forests models) in a series of experiments based on naturalistic driving data to investigate the relationship between changes in driving behaviors, space and performance and the risk of MCI/dementia. The classifiers with driving variables produce much higher F_1_ scores than those with age and other demographic characteristics. Safe driving requires essential cognitive and physical functions and perceptual motor skills. As a complex task involving dynamic cognitive processes, naturalistic driving behavior features can be used as comprehensive and reliable phenotypic markers to detect preclinical AD, MCI and dementia [15].

A notable limitation of this study is the modest number of incident MCI and dementia cases. As a result, we included driving behavior features across all the study participants in the classification algorithms without specifying which records belong to whom, or considering time-series sequences of those driving features. It is also noteworthy that MCI is not necessarily a prodromal stage of dementia, although many MCI cases do progress to dementia, with an annual conversion rate of about 12% in the general population [16]. As the LongROAD project was designed to study aging and driving safety, the research protocol did not include collecting detailed diagnostic data related to MCI and dementia, such as neurological tests and imaging biomarkers. Future research should develop prediction models for MCI and dementia separately and for progression from MCI to dementia. Another limitation of the study is that the random forests models were trained and tested with oversampled MCI/dementia data points. Although rebalancing the dataset is necessary for avoiding training bias, results from the random forest models may not be entirely generalizable to the real-world setting as the actual dataset is highly imbalanced.

Nevertheless, the preliminary findings indicate that the performance of the random forest model based on basic demographic characteristics and driving behavior features is excellent, with a F_1_ score of 0.88 and an area under the receiver operating characteristic curve of 0.90. When additional follow-up data become available, we will use individual-level longitudinal driving data to develop a personalized time-dependent classifier to predict the risk of MCI/dementia for each study participant. If the high accuracy of the classifier is confirmed, the algorithm based on driving behavior features along with basic demographic characteristics could be incorporated into a smartphone app and other devices for early detection of MCI and dementia in older adult drivers.

## Figures and Tables

**Table 1:** Variable definitions and statistics.

Variable		Name	Definition	Statistics			
				Min	Max	Mean	SD
Diagnosis_labels		MCI/Dementia	One’s disease level in a month (0-Healthy; 1-Mild Cognitive Impairment (MCI)/Dementia/Alzheimer’s	0	1	-	-
Demographic characteristics							
	Age in years	Age	Age at enrollment	65	79	71.1	4.1
	Sex	Sex	Male; Female	NA	NA	NA	NA
	Race/Ethnicity	Race	Alaska Native, Native Hawaiian, Pacific Islander; American Indian, Asian; Black (non-Hispanic); White (non-Hispanic); Hispanic; Other	NA	NA	NA	NA
	Education	Education	Associate degree; Bachelor degree; Master, professional, or doctoral degree; Some college but no degree; Vocational, technical, business, or trade school (beyond high school level); Other	NA	NA	NA	NA
Driving variables							
1	Miles_n	Miles	Total number of miles driven in month	0	15,783	762.2	587.8
2	Trips	Trips	Total number of trips in month	1	2341	115.8	64.6
3	TripsLt15Miles	No. trips < 15 miles of home	Number of trips traveled in month within 15 miles of home	0	1953	95.5	58.4
4	PercentDistLt15Miles_n	% trip < 15 miles of home	Percent of trips traveled in month within 15 miles of home	0.0	100.0	64.9	28.9
5	TripsLt25Miles	No. trips < 25 miles of home	Number of trips traveled in month within 25 miles of home	0	1953	101.7	59.7
6	PercentDistLt25Miles_n	% trip < 25 miles of home	Percent of trips traveled in month within 25 miles of home	0.0	100.0	76.5	26.4
7	MilesPerTrip_n	Miles per trip	Total number of miles driven in month divided by total number of trips in month	0.0	74.5	6.7	4.1
8	MinutesPerTrip_n	Minutes per trip	Total driving minutes in month divided by total number of trips in month	0.1	137.7	14.9	5.9
9	TripMinutes_n	Total trip minutes	Total minutes of driving in month	0.1	16,645.0	1633.4	1083.6
10	TripsInDay	No. trips during day	Number of trips in month not classified as nighttime	0	1279	107.2	57.4
11	PercentTripsInDay_n	% trips during day	Percent of trips in month not classified as nighttime	0.0	100.0	93.1	8.0
12	TripsAMPeak	No. trips in AM peak	Number of trips in month during 7–9 AM on weekdays	0	167	8.6	9.4
13	PercentTripsAMPeak_n	% trips in AM peak	Percent of trips in month during 7–9 AM on weekdays	0.0	100.0	7.3	6.9
14	TripsAtNight	No. trips at night	Number of trips during which at least 80% of a trip was during nightime in month (Nightime was defined as civil twilight or a solar angle greater than 96 deg)	0	1143	8.7	16.6
15	PercentTripsAtNight_n	% trips at night	Percent of trips during which at least 80% of a trip was during nightime in month (Nightime was defined as civil twilight or a solar angle greater than 96 deg)	0.0	100.0	6.9	8.0
16	TripsPMPeak	No. trips in PM peak	Number of trips in month during 4–6PM on weekdays	0	150	10.9	9.5
17	PercentTripsPMPeak_n	% trips in PM peak	Percent of trips in month during 4–6PM on weekdays	0.0	100.0	9.3	6.7
18	LeftTurnCount	No. left turns	Number of left turns made in month	0	2592	261.6	159.9
19	RightTurnCount	No.right turns	Number of right turns made in month	0	2751	242.6	150.1
20	RightToLeftTurnRatio_n	Right to left turn ratio	Ratio of all right-hand to left-hand turning events for a driver in a month	0.0	7.0	0.9	0.2
21	TripsVgt60	No. trips on high speed roads	Number of trips in month where 20% of distance travelled was at a speed of 60 MPH or greater	0	226	13.9	15.0
22	PercentTripsVgt60_n	% trip on high speed roads	Percent of trips in month where 20% of distance travelled was at a speed of 60 MPH or greater	0.0	12.6	100.0	12.3
23	SpeedGt80mphCount	No. speeding events	Number speeding events in month (speed > 80 MPH sustained for at least 8 s)	0	3300	7.3	31.3
24	DecelCntLtN3pt5Mps2	No. hard braking events with deceleration rates ≥ 0.35 g	Number of events with a deceleration rate ≥ 0.35 g in a month	0	1112	3.8	8.2
25	DecelCntLtN4pt0Mps2	No. hard braking events with deceleration rates ≥ 0.40 g	Number of events with a deceleration rate ≥ 0.4 g in a month	0	734	0.9	4.2
26	DecelCntLtN7pt5Mps2	No. hard braking events ≥ 0.75 g	Number of events with a deceleration rate ≥ 0.75 g in a month	0	24	0.005	0.2
27	TripChains	Trip chains	Number of trip chains in month (Note: chain is a series of trips starting and ending at home)	0	180	8.2	7.8
28	MilesPerChain_n	Miles per chain	Total miles of chains in month divided by total number of trip chains in month	0.00	4273.3	100.7	121.1
29	MinutesPerChain_n	Minutes per chain	Total driving minutes for chains divided by total number of trip chains in month	0.0	6606.3	222.0	219.8

NA, not applicable.

**Table 2:** Performance of random forests models with different covariates in predicting incident mild cognitive impairment or dementia.

Model	Covariates	Accuracy	Precision or PPV	Recall or Sensitivity	Specificity	NPV	F1 Score	AUC	Out-of-Bag Error Rate		Confusion Matrix		
											Predicted	Observed	
									%	SD		0	1
1	Age only	0.46	1.00	0.06	1.00	0.45	0.11	0.56	6.00	0.01	0	77	96
											1	0	6
2	Age, sex, race/ethnicity, and education	0.53	1.00	0.17	1.00	0.48	0.29	0.64			0	77	85
									4.27	0.01	1	0	17
3	Driving variables only	0.66	0.79	0.56	0.81	0.58	0.66	0.76	2.60	0.75	0	62	45
											1	12	57
4	Age and driving variables	0.80	0.89	0.74	0.88	0.72	0.81	0.91	2.14	0.50	0	68	27
											1	9	75
5	Age, sex, race/ethnicity, education and driving variables	0.86	0.86	0.90	0.81	0.86	0.88	0.90	2.07	0.57	0	62	10
											1	15	92

PPV, positive predictive value; NPV, negative predictive value; AUC, area under the receiver operating characteristic curve.

## Data Availability

Restrictions apply to the availability of these data. Data are available from the author with permission from the AAA Foundation for Traffic Safety and upon execution of a data use agreement.
